# Ethylene is critical to the maintenance of primary root growth and Fe homeostasis under Fe stress in *Arabidopsis*

**DOI:** 10.1093/jxb/erv005

**Published:** 2015-02-22

**Authors:** Guangjie Li, Weifeng Xu, Herbert J. Kronzucker, Weiming Shi

**Affiliations:** ^1^State Key Laboratory of Soil and Sustainable Agriculture, Institute of Soil Science, Chinese Academy of Sciences, No. 71 East Beijing Road, Nanjing 210008, PR China; ^2^Department of Biological Sciences, University of Toronto, 1265 Military Trail, Toronto, Ontario M1C 1A4, Canada

**Keywords:** *Arabidopsis*, ethylene, Fe homeostasis, Fe toxicity, primary root growth.

## Abstract

Iron (Fe) is an essential microelement but is highly toxic when in excess. The response of plant roots to Fe toxicity and the nature of the regulatory pathways engaged are poorly understood. Here, we examined the response to excess Fe exposure in *Arabidopsis* wild type and ethylene mutants with a focus on primary root growth and the role of ethylene. We showed that excess Fe arrested primary root growth by decreasing both cell elongation and division, and principally resulteds from direct external Fe contact at the root tip. Pronounced ethylene, but not abscisic acid, evolution was associated with excess Fe exposure. Ethylene antagonists intensified root growth inhibition in the wild type, while the inhibition was significantly reduced in ethylene-overproduction mutants. We showed that ethylene plays a positive role in tissue Fe homeostasis, even in the absence of iron-plaque formation. Ethylene reduced Fe concentrations in the stele, xylem, and shoot. Furthermore, ethylene increased the expression of genes encoding Fe-sequestering ferritins. Additionally, ethylene significantly enhanced root K^+^ status and upregulated K^+^-transporter (HAK5) expression. Our findings highlight the important role of ethylene in tissue Fe and K homeostasis and primary root growth under Fe stress in *Arabidopsis*.

## Highlight

We propose that ethylene is involved in the regulation of excess Fe tolerance by maintenance of tissue Fe and K homeostasis in *Arabidopsis*.

## Introduction

Iron (Fe) is an essential element for plants, but excessive presence of this element in soils and subsequent toxicity is common, especially in acidic and water-logged soils (Connolly and Guerinot, 2002). Yield reductions of 10–100% have been reported in the case of the world’s leading crop species, rice, and indeed Fe toxicity is considered one of the most formidable research and management challenges in rice cultivation (Becker and Asch, 2005). It has also been recorded in other important crops such as wheat (Khabaz-Saberi *et al.*, 2010, 2012). Our understanding of the mechanisms underlying Fe toxicity remains sparse but is essential in confronting this significant agronomic problem.

The primary mechanisms underlying Fe toxicity in plants remain poorly described. It is well established that of the two oxidation-state species of Fe, Fe^2+^ and Fe^3+^, the former is more persistent in acidic soils, most readily absorbed by plants roots, and most detrimental to plant metabolism on account of its influence on redox chemistry. The key chemical reactions proceeding intracellularly, and leading to oxidative tissue damage, are known as the Fenton reactions, which proceed in two steps, each leading to the production of strong oxidants, and, in turn, setting the stage for ‘run-away’ oxidative chain reactions:

$${\text{Fe}}^{\text{2}+}+{\text{H}}_{2}{\text{O}}_{2}\to {\text{Fe}}^{\text{3}+}+{\text{HO}}^{•}+\text{OH}$$

$${\text{Fe}}^{\text{3}+}+{\text{H}}_{2}{\text{O}}_{2}\to {\text{Fe}}^{\text{2}+}+{\text{HOO}}^{•}+{\text{H}}^{+}$$

Accumulation of excess free Fe^2+^ in tissues must thus be prevented. Several strategies to minimize free Fe^2+^ build-up in plants have been proposed, such as the exclusion of Fe^2+^ at the root level, by oxidation and formation of ‘iron plaques’ in the rhizosphere, ion selectivity at the uptake step (Chen *et al*., 1980*a*, *b*), sequestration in vacuoles and the apoplast, occlusion in ferritin proteins (Audebert and Sahrawat, 2000; Pich *et al.*, 2001; Majerus *et al.*, 2009), and enzymatic detoxification in the symplast (Majerus *et al.*, 2007*b*, 2009). Furthermore, a common symptom of Fe toxicity is a general suppression of tissue cation levels, such as those of K^+^, Ca^2+^, and Mg^2+^ (Becker and Asch, 2005; Çelik *et al.*, 2010). Therefore, maintenance of tissue Fe^2+^ and other vital cations must be considered key to tolerance to Fe toxicity.

Several studies have linked the stress hormone ethylene to Fe toxicity (Peng and Yamauchi, 1993; Becker and Asch, 2005; Majerus *et al.*, 2007*a*). Ethylene production has been shown to increase under excess Fe (Peng and Yamauchi, 1993; Yamauchi and Peng, 1995). In rice, increased ethylene production has been linked to differential Fe tolerance of various cultivars, and, more specifically, to iron-plaque formation on the root surface in connection with ethylene-induced aerenchyma differentiation (Majerus *et al.*, 2007*a*; Harahap *et al.*, 2014). In addition, there is also evidence suggesting that abscisic acid (ABA) may play a role in Fe tolerance (Majerus *et al.*, 2009). As with ethylene, abscisic acid concentrations have been shown to increase in rice upon exposure to excess iron, and application of an inhibitor of ABA biosynthesis (fluridone, FLU) led to increased iron concentrations in the shoot (Majerus *et al.*, 2009). However, the detailed characteristics and more specific mechanisms of ethylene and ABA responses under Fe stress remain largely unclear.

Increasing evidence points to the importance of ethylene in promoting growth, particularly in the response to abiotic stresses (Pierik *et al.*, 2006). Recent studies have demonstrated that ethylene plays an important role in regulating root growth tolerance to selenite and sodium stresses (Cao *et al.*, 2007; Lehotai *et al.*, 2012). Similarly, ABA plays an important role in regulating root growth (Xu *et al.*, 2013). For instance, increased accumulation of ABA is required for maintaining primary root growth during aluminium (Al) exposure (Hou *et al.*, 2010). Inhibition of primary root growth is one of the earliest symptoms exhibited in response to excess Fe levels (Yoshida, 1981; Becker and Asch, 2005; Fageria *et al.*, 2008; Zhang *et al.*, 2011, 2012). Yamauchi and Peng (1995) reported root-growth retardation and a reduction and shortening of roots under Fe exposure. Root growth decreased with increasing external Fe concentration (Li *et al.*, 2012). Zhang *et al.* (2011, 2012) proposed that the root tip maybe the main action site for Fe resistance. In addition, it has been shown that the application of potassium (K) can ameliorate Fe-inhibited root growth (Li *et al.*, 2001). Although ethylene and ABA signals have been implicated as important for root growth, it is not known how ethylene and ABA are involved in the response of primary root growth to Fe stress.

In this study, we employed *Arabidopsis* wild type (WT) and ABA- and ethylene-sensitive mutants, to explore the primary root growth response to Fe toxicity, and to elucidate the roles of ethylene and ABA. Potential mechanisms involved in the stress response to Fe are discussed.

## Materials and methods

### Plant materials and growth conditions

Seedlings of the following lines were used in this study: *Arabidopsis thaliana* ecotype Columbia-0 (Col-0); the mutants *eto1-1* (At3g51770), *ctr1-1* (At5g03730), *aba3-1* (At1g16540), *aba2-3* (At1g52340), *abi1-1* (At4g26080), *eto2-1* (At5g65800), *etr1-3* (At1g66340), and *eto1-1/etr1-3* in the Col-0 background, and the transgenic lines *CycB1::GUS* (Colon-Carmona *et al.*, 1999), *QC25::GUS* (Sabatini *et al.*, 2003), and *AtACS7::GUS* (Wang *et al.*, 2005). Seeds were surface sterilized and cold treated at 4 °C for 48h before being sown on standard growth medium. The standard growth medium has been described previously (Li *et al.*, 2010) and was composed of 2mM KH_2_PO_4_, 5mM NaNO_3,_ 2mM MgSO_4_, 1mM CaCl_2_, 50 μM Fe-EDTA, 50 μM H_3_BO_3_, 12 μM MnSO_4_, 1 μM ZnC1_2_, 1 μM CuSO_4_, 0.2 μM Na_2_MoO_4_, 1% sucrose, 0.5g l^–1^ MES, and 0.8% agar (adjusted to pH 5.7 with 1M NaOH). The day of sowing was considered as d 0. Seedlings were grown, oriented vertically on the surface of the culture plates in a growth chamber and set to a 16h light/8h dark photoperiod, an irradiance of 100 μmol m^−2^ s^−1^, and a temperature of 23±1 °C.

### Whole-root and localized-root supply of Fe-EDTA to the primary root

Root Fe treatments were applied as follows. Fe was supplied as Fe-EDTA (FeSO_4_.7H_2_O plus EDTA, 1:1 molar ratio). The design for whole-root versus supply of Fe-EDTA to only the root tip or the mature zone of the primary root is shown in Fig. 2A, and is briefly described here: segmented agar plates (13×13cm) were separated into upper and bottom parts with a 3mm air gap (Zhang and Forde, 1998) using movable glass strips 3mm in width. Normal growth medium (control medium, pH 5.7) without Fe-EDTA was poured into the upper part, and control medium containing various concentrations of Fe-EDTA was poured into the bottom part. For ‘root-supplied Fe’ plants, the whole root was in contact with the bottom medium after seedling transfer. Plates on which only the primary root tip of the seedlings (~2mm) was in contact with the bottom of the medium are referred to as ‘root-tip-supplied Fe’ plants. In order to perform localized applications of nutrients to the mature zone of the primary root, the segmented plates were separated into three parts: upper, middle, and bottom. The medium containing Fe-EDTA was only poured into the middle part of the plates. For the mature root zone treated with Fe-EDTA, 6-d-old seedlings (with primary roots long enough to cross the middle agar region) were transferred, while others were 5-d-old seedlings, and the treatment length was all 5 d. To study the effect of ethylene precursor or inhibitors and ABA or ABA inhibitor, the bottom medium was supplemented with various concentrations of Fe-EDTA plus the indicated concentrations of ABA, FLU, 1-aminocyclopropane-1-carboxylic acid (ACC), aminoethoxyvinylglycine (AVG), or amino-oxyacetic acid (AOA). All chemicals were obtained from Sigma-Aldrich. To study the effect of K^+^ on root elongation, the bottom medium only was supplied with various concentrations of K^+^.

Because Fe availability and toxicity are strongly pH dependent (Becker and Asch, 2005), and the pH dependence of root growth itself (Koyama *et al.*, 2001; Kinraide, 2003; Yang *et al.*, 2005) must be controlled for, we also conducted a separate analysis of *Arabidopsis* primary root growth on a pH gradient both with and without excess Fe. Based on preliminary results showing no effect on root elongation in no-Fe controls, but a significant inhibition in Fe treatments at pH 5.3 (see Supplementary Fig. S1 at *JXB* online), we selected this pH in further examinations (established in the agar plate bottom parts).

### Measurement of root length, lateral root number, and chlorophyll content

Roots on the agar surface were sampled. The lengths of primary roots of individual seedlings were measured directly with ImageJ software from digital images captured with a Canon G7 camera. Primary root elongation was defined as the length of the root parts newly grown after treatment. The number of mature lateral roots was counted under a dissecting microscope after an additional 5 d of growth. Chlorophyll content was assayed according to the method of Wintermans and de Mots (1965).

### Microscopic analysis of cell development in roots

Histochemical analysis of β-glucuronidase (GUS) reporter enzyme activity was performed according to the procedure by Weigel and Glazebrook (2002). The length, area, and intensity of the GUS-stained zone were analysed using ImageJ software from digital images captured with a Canon G7 camera. Roots were mounted directly in double-distilled water to avoid cell shrinkage (Li *et al.*, 2010). Measurements were made of the length from the first elongated cell to the first root hair, the meristem length from the quiescent centre (QC) to the first elongated cell, and the final cell length as indicated by the cortex cell length at the mature zone on newly grown parts of the primary root. All images were obtained using an Olympus BX51 microscope equipped with differential interference contrast (DIC) optics and an Olympus DP71 camera. The length of a single cell or tissue in the primary root was analysed with the software Image-Pro Express version 5.1 (Media Cybernetics, Bethesda, MD, USA).

### Ethylene measurements

After seedling exposure to various concentrations of Fe-EDTA for 4 d, roots were weighed separately and put into 5ml gas-tight vials containing 1ml of agar medium (0.7% agar). Headspace samples (1ml) were withdrawn and analysed using a GC-6850 gas chromatograph (Agilent Technologies Japan), which was equipped with a flame ionization detector.

### *In vivo* staining of ferric and ferrous Fe

For localization of ferric Fe (Fe^3+^), *Arabidopsis* roots were rinsed three times with 10mM EDTA and vacuum infiltrated with Perls’ stain solution (equal volumes of 4%, v/v, HCl and 4%, w/v, potassium ferrocyanide) (Stacey *et al.*, 2008) for 15min. Samples were incubated for another 15min in the stain solution and rinsed three times with water. Localization of ferrous Fe (Fe^2+^) was analysed as described by Engel *et al.* (2012). Briefly, roots were excised, rinsed three times with 10mM EDTA, and then rinsed three times with ultrapure water (18.2 Milli-Q cm^–1^). The samples were transferred to 5mM 2,2′-bipyridine in 50mM Tris/acetate buffer at pH 5.6 and incubated for durations of 6h under permanent light (300 μmol m^–2^ s^–1^) at 30 °C. Localization of Fe^2+^ and Fe^3+^ was observed and imaged using an Olympus BX51 microscope equipped with DIC optics and an Olympus DP71 camera.

### Characterization of root plaques by scanning electron microscopy (SEM)/energy-dispersive X-ray (EDX)

Five-day-old WT and *eto1-1* seedlings were transferred to control or excess Fe medium with or without AVG for an additional 5 d of growth. Roots were washed gently with deionzied water three times, and the materials formed were observed on drying roots by SEM (Hitachi S4200, operated at 15kV). The elemental composition of the materials on the roots was examined by EDX attached to the SEM, according to the method of Mi *et al.* (2013).

### Mineral analysis

Five-day-old WT and *eto1-1* seedlings were transferred to control or excess Fe medium with or without AVG for an additional 5 d of growth and then harvested. For mineral analyses, the roots and shoots of the seedlings were dried at 75 °C prior to analysis, and samples were digested with HNO_3_ and subjected to inductively coupled plasma atomic emission spectroscopy (ICP-AES) (IRIS Advantage, Thermo Electron, USA).

### Quantitative real-time reverse transcription PCR (qRT-PCR) analysis

qRT-PCR was carried out according to the method of Li *et al.* (2013). Total RNA was extracted from *Arabidopsis* roots. Gene sequences were available at the National Center for Biotechnology Information, and gene-specific primers for qRT-PCR were designed and identified using Primer 5 software and ordinary PCR, respectively (Supplementary Fig. S11A and Table S1 at *JXB* online). CBP20 (nuclear-encoded cap-binding protein) was used as the housekeeping gene, and relative RNA abundance was normalized to the CBP20 internal control ([mRNA]_gene_/[mRNA]_CBP20_).

### Statistical and graphical analyses

For all experiments, data were statistically analysed using the SPSS 13.0 program (SPSS, Chicago, IL, USA.). Details are as presented in the figure legends. Graphs were produced using Origin 8.0. All graphs and images were prepared using Adobe Photoshop 7.0.

## Results

### Root-tip contact with excess Fe is essential for inhibition of *Arabidopsis* primary root growth

**Fig. 1.**
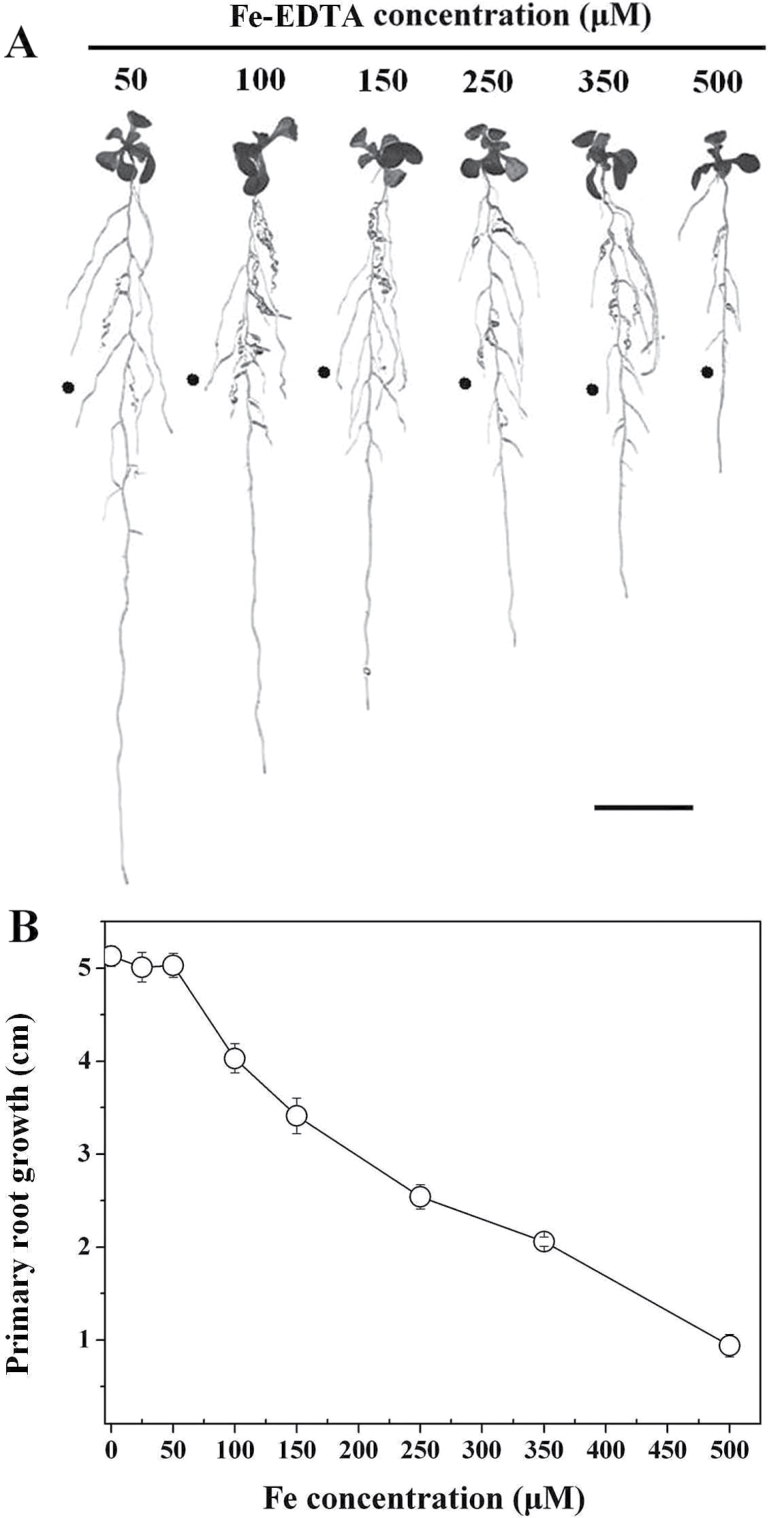
Inhibitory effect on the growth of primary root in *Arabidopsis* (Col-0) when roots are supplied with Fe-EDTA. (A) Photograph of representative seedlings after 5 d of vertical growth on varying Fe concentrations. The filled circle indicates the positions of primary root tips at the time of transfer to different treatment media. Bar, 1cm. (B) Primary root growth of 5-d-old WT exposed to serial concentrations of Fe-EDTA for 5 d. Data represent means of eight or more plants±standard error (SE).

To analyse the effect of Fe toxicity on primary root growth in 5 d treatments, *Arabidopsis* roots were allowed to come into contact with varying concentrations of Fe-EDTA. Root-supplied Fe from 0 to 50 μM did not significantly affect primary root growth. However, at Fe concentrations at and above 100 μM, elongation was potently inhibited, and the degree of inhibition was positively dependent on Fe concentration (Fig. 1). By contrast, lateral root number and chlorophyll content were more resistant to higher Fe concentrations (Supplementary Fig. S2 at *JXB* online). At 50 μM Fe, seedlings showed maximum primary root growth (Fig. 1) coupled to green leaves and healthy growth overall (Supplementary Fig. S2). Therefore, 50 μM Fe was used as the control in further experiments.

**Fig. 2.**
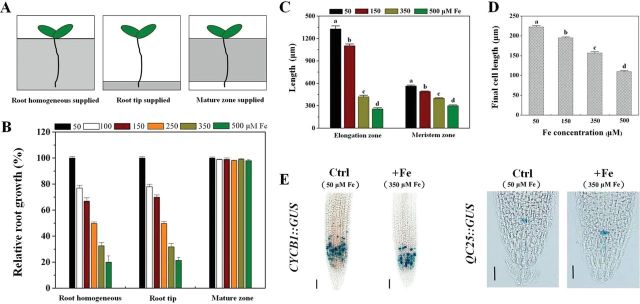
Effect of excess Fe on cell elongation and cell division of primary root tips. (A) Schematic diagram of experimental setup for applying whole-root and localized excess Fe treatments to *Arabidopsis* roots. White sections indicate the basal growth medium without Fe and grey areas indicate the Fe-enriched medium. (B) Effect of applying serial concentrations of Fe to different parts of the primary root on primary root growth; 100% corresponds to 5.2±0.17cm in the root-supplied Fe treatment, 5.1±0.12cm in the root-tip-supplied Fe treatment, and 6.1±0.13cm in the mature-zone-supplied Fe treatment. The values represent the means±SE of five or more plants. (C) Root elongation and meristem zone sizes in 5-d-old *Arabidopsis* seedlings treated with different Fe concentrations for 5 d. Bars represent means±SE of 10 or more plants. Different letters represent statistically different means at *P*<0.05 ([one-way analysis of variance (ANOVA) with Duncan post-hoc test]. (D) Comparison of the effect of Fe on the final cell length in primary roots in the root-supplied Fe treatment (5 d). Values represent means±SE of more than 80 cells from no fewer than seven independent plants. Different letters represent statistically different means at *P*<0.05 level (one-way ANOVA analysis with Duncan post-hoc test). (E) Effect of temporal root-supplied excess Fe on the expression of *CycB1::GUS* and *QC25::GUS*, after staining for 16h. One representative sample for each experiment is shown. Bars, 50 μm.

Given the strong impact of excess Fe exposure on primary root growth, we proceeded to determine if this was a localized or systemic response. We first tested the growth response of primary roots to localized Fe supply (Fig. 2A), and found that the inhibitory effect was identical whether Fe was applied to the whole root or only the tip (Fig. 2B). In a further experiment, no growth inhibition occurred when the root body was in contact with Fe but the root tip did not touch the Fe-containing medium, even when Fe concentration was raised to 500 μM (Fig. 2B). These observations demonstrated a highly localized response to excess Fe in the inhibition of root growth phenotype.

We further observed that the root-elongation zone was markedly reduced in response to excess Fe treatment compared with the control (for example, 68% reduction at 350 μM Fe) (Fig. 2C), indicating either a reduced capacity for cell expansion in the elongation zone and/or reduced cell division in the meristem. To address these two possibilities, we first examined the length of differentiated cells just above the elongation zone, representing root-cell expansion (Li *et al.*, 2010). The average length of differentiated cells in seedlings subjected to excess Fe showed dose-dependent inhibition (Fig. 2D). The capacity for cell division in the root apex was then measured. Meristem length was reduced at and above 150 μM Fe, and decreased linearly as medium Fe concentration increased further (Fig. 2C). The *CycB1::GUS* reporter was further used to monitor cell-cycle activity, specifically the G_2_-to-M transition during the cell cycle (Colon-Carmona *et al.*, 1999). Even though root-supplied Fe had no significant effect on the intensity of expression of *CycB1::GUS* in the root meristem compared with the control (Fig. 2E and Supplementary Fig. S5C at *JXB* online), analysis of the extent and area of the GUS-stained region of the root tip indicated that the mitotically active zone was significantly reduced (*P<*0.05) (Supplementary Fig. S5A, B). We further examined the expression of *QC25*, an established QC marker. Interestingly, compared with the control, expression of *QC25::GUS* in the root tip under excess Fe was slightly increased (*P<*0.05) (Fig. 2E and Supplementary Fig. S5D).

### ABA-mediated signalling is not required for the inhibitory action of Fe on primary root growth

**Fig. 3.**
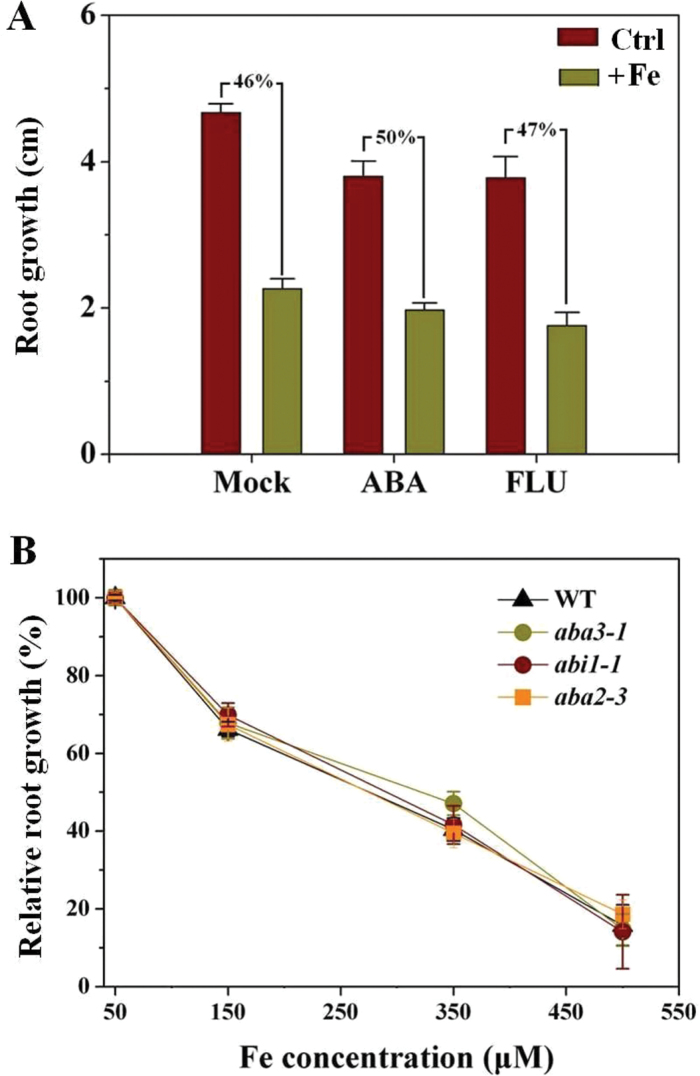
ABA-mediated signalling is not required for the inhibitory action of Fe on primary root growth. Five-day-old WT, *abi1-1*, *aba3-1*, and *aba2-3* seedlings were transferred to medium, and roots were supplemented with 350 μM Fe alone or in combination with 3 μM ABA or 0.1 μM FLU for 5 d, after which primary root growth was quantiﬁed. (A) Effect of ABA or FLU on primary root growth in WT seedlings when roots are supplied with excess Fe. Values are means±SE (*n*≥6). (B) Effect of varying concentrations of Fe on primary root growth in WT and ABA mutant seedlings. Values are means±SE (*n*≥5). Primary root growth in WT, *abi1-1*, *aba3-1*, and *aba2-3* in the control were 5.2±0.1, 4.66±0.14, 3.92±0.2, and 4.3±0.12cm, respectively.

ABA plays an important role in regulating root growth under stress (Xu *et al.*, 2013), including under high-Fe exposure (Majerus *et al.*, 2009), and thus had to be examined here. Specifically, the involvement of ABA in the regulation of primary root growth under excess Fe has not been investigated. For this purpose, the effects of ABA and FLU, an inhibitor of ABA biosynthesis, on primary root growth under conditions under both control and excess Fe conditions were examined. However, neither ABA nor FLU affected the inhibition of primary root growth by excess Fe significantly (Fig. 3A). Furthermore, ABA mutants, including *abi1-1* (ABA-insensitive), *aba3-1*, and *aba2-3* (ABA-deficient), were used to test whether inhibition of primary root growth under Fe stress involved ABA mediation. The mutants and WT seedlings were exposed to varying concentrations of Fe continuously for 5 d and the lengths of newly grown roots were analysed statistically (Fig. 3B). Fe had a similar impact on inhibition of root growth in *abi1-1*, *aba3-1*, *aba2-3*, and WT plants.

### Excess Fe-induced root ethylene accumulation contributes to primary root-growth tolerance

**Fig. 4.**
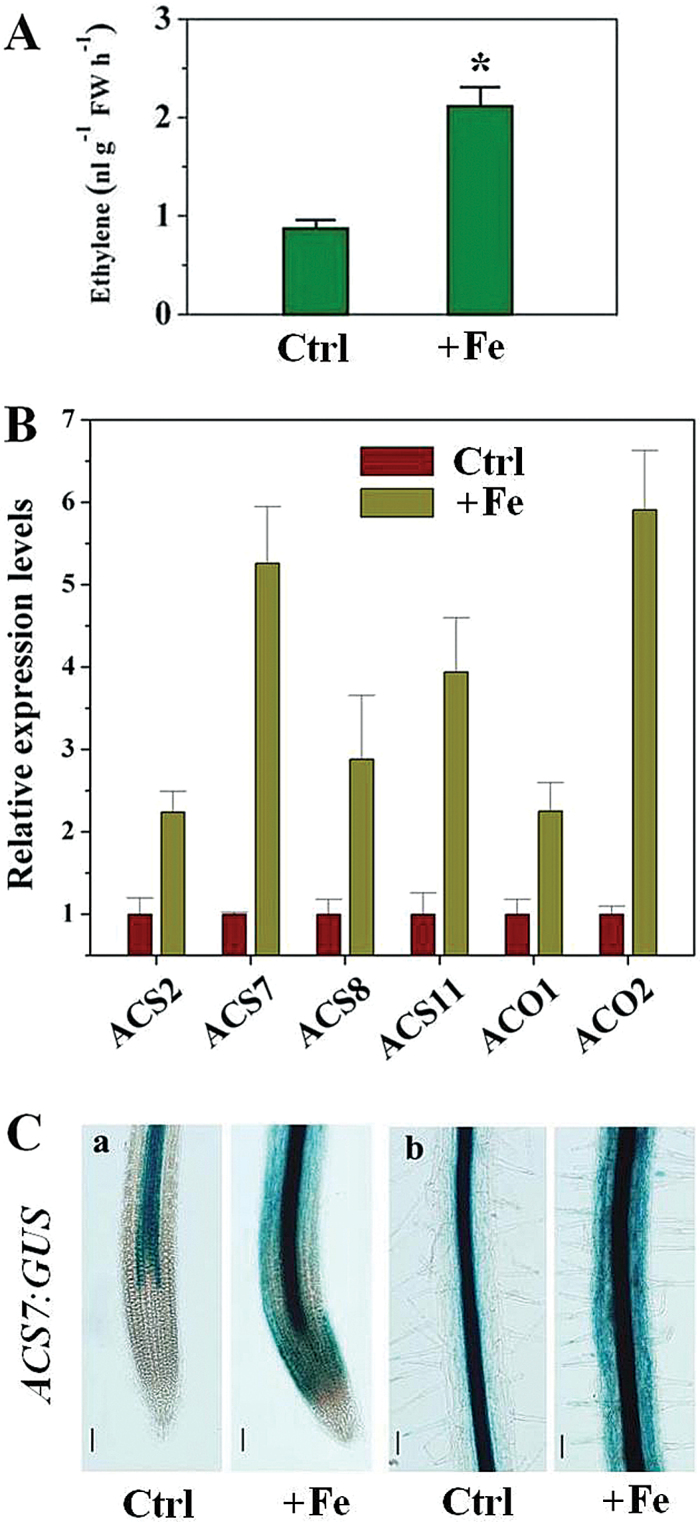
Effect of excess Fe on ethylene response in WT *Arabidopsis* seedlings. (A) Ethylene evolution in *Arabidopsis* root. Seedlings at 4 d after germination were exposed to 350 μM in the roots for 4 d, and ethylene evolution was determined. Values are means±SE of three replicates. Asterisks indicate statistical differences between control (Ctrl) and excess Fe treatment (+Fe) conditions (independent samples *t*-test, *P<0.05). (B) Effect of excess Fe on expression of root ACS and ACO. Expression of root ACS and ACO were determined by quantitative real-time PCR after exposure of 5-d-old WT seedlings to 350 μM Fe for 6h. Values are means±SE of three replicates. (C) Activity of *AtACS7::GUS* in *Arabidopsis* root tissue. Seedlings at 5 d after germination were exposed to 350 μM Fe in the roots for 2 d, and then *ACS7::GUS* activity was determined. One representative sample from each treatment (seven plants) is shown. (a) Primary root apex; (b) stele of primary root. Ctrl, control. Bars, 50 μm.

To elucidate the relationship between ethylene accumulation and Fe tolerance of root growth, we first measured ethylene production under Fe stress by use of gas chromatography. Excess Fe significantly increased ethylene levels in roots (Fig. 4A). As ACC synthase (ACS) and ACC oxidase (ACO) are pivotal enzymes in the ethylene biosynthetic pathway of plants, to detect whether the observed induction of ethylene production by excess Fe was due to regulation of expression of the genes encoding ACS and ACO in roots, four genes for ACS, *AtACS2*, *AtACS7*, *AtACS8*, and *AtACS11*, and two for ACO, *AtACO1*and *AtACO2*, were examined, using qRT-PCR. All *ACS* genes responded with increased transcript abundance following 6h treatment with excess Fe, and expression of *AtACO1* and *AtACO2* was also rapidly upregulated in response to excess Fe after 6h (Fig. 4B). The expression levels of *AtACS7::GUS*, in which the GUS reporter gene is driven by an *AtACS7* promoter, were also tested, and excess Fe induced a marked increase of *AtACS7::GUS* expression from the meristematic to the mature zone of roots, compared with the control (Fig. 4C). We also analysed the expression of the ethylene reporter *EBS::GUS* (an ethylene reporter construct in which the GUS reporter gene is driven by a synthetic EIN3-responsive promoter) in the root (Supplementary Fig. S3 at *JXB* online). On excess Fe-treated medium, *EBS::GUS* expression was enhanced markedly from the elongation zone to the mature zone of the roots, compared with that of the control (Supplementary Fig. S3).

**Fig. 5.**
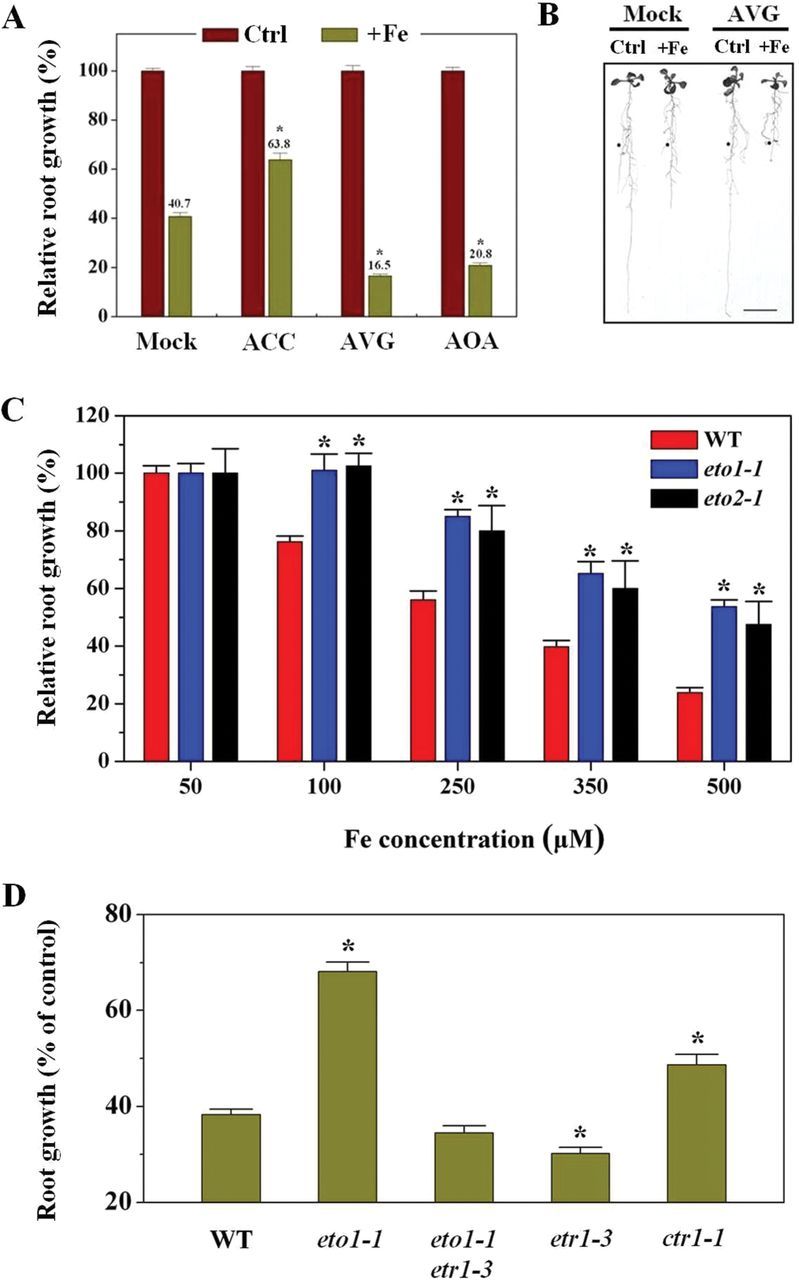
Enhanced ethylene evolution contributes to primary root growth tolerance to excess Fe. Five-day-old WT, *eto1-1*, *eto2-1*, *etr1-3*, *ctr1-1*, and *eto1-1etr1-3* seedlings were transferred to medium, and roots were supplemented with 350 μM Fe alone or in combination with 0.2 μM ACC, 1 μM AVG,or 50 μM AOA for 5 d, after which primary root growth was quantiﬁed. (A) Effect of root-supplied ethylene precursor (ACC) or inhibitors (AVG and AOA) on primary root growth of WT *Arabidopsis* seedlings grown in 350 μM Fe treatment medium. Values are means±SE (*n*≥ 6). Asterisks indicate statistical differences between the mock and treatment under excess Fe condition (independent samples *t*-test, *P<0.05). (B) Photograph of representative seedlings after 5 d of vertical growth in medium with roots supplemented with 350 μM Fe alone or in combination with 1 μM AVG. Filled circles indicate the positions of primary root tips at the time of transfer to different treatment media. Bars, 1cm. (C) Effect of excess Fe on primary root growth in WT and ethylene-overproducing mutants *eto1-1* and *eto2-1*. Data are from one of three experiments. Values are means±SE (*n*≥5). Asterisks indicate statistical differences between the WT and mutants in the same growth conditions (independent samples *t*-test, *P<0.05). Primary root growth in WT, *eto1-1*, and *eto2-1* in the control were 5.2±0.24, 2.97±0.2, and 3.14±0.28cm, respectively. (D) Relative root growth of WT, *eto1-1*, *etr1-3*, *ctr1-1*, and *eto1-1etr1-3* seedlings on excess Fe treatment compared with the control. Values are the means±SE (*n*=5). Asterisks indicate statistical differences between the WT and mutants under excess Fe condition (independent samples *t*-test, *P<0.05). Primary root growth in WT, *eto1-1*, *etr1-3*, *ctr1-1*, and *eto1-1etr1-3* in control were 5.14±0.32, 2.48±0.3, 5.0±0.12, 2.34±0.15, and 5.1±0.21cm, respectively.

To investigate the possible role of ethylene in Fe-induced inhibition of primary root growth, we investigated the effects of ACC (an ethylene precursor), AVG, and AOA (inhibitors of ethylene biosynthesis) on primary root growth under conditions of both control and excess Fe. Supplementation with ACC effectively reversed the decreased rate of primary root growth seen under excess Fe, compared with the mock condition (36.2 and 59.3% inhibition, respectively; Fig. 5A). However, AVG and AOA aggravated the Fe-induced inhibition of primary root growth (Fig. 5A, B). A genetic approach was further adopted, using an ethylene-overproduction mutant *eto1-1* (Alonso and Stepanova, 2004). Exposure of the *eto1-1* mutant to varying Fe concentrations led to less inhibition of root growth than in WT, with elongation being reduced by ~60 and ~35% in WT and *eto1-1* plants, respectively, upon exposure to 350 μM Fe (Fig. 5C). We next determined if other mutations conferring increased ethylene levels also led to increased primary root growth tolerance to excess Fe and if application of the ethylene biosynthesis inhibitor AVG would suppress *eto1-1* mutant root growth tolerant phenotypes. We found that *eto2-1*, a gain-of-function *ACS5* mutant allele of *ETO2*, which confers increased ethylene production (Wang *et al.*, 2004), also displayed increased root-growth tolerance compared with WT (Fig. 5C). Meanwhile, supplementation with AVG prevented root growth tolerance in association with elevated ethylene levels in *eto1-1* seedlings exposed to excess Fe (Supplementary Fig. S4 at *JXB* online).

As ethylene is known to activate downstream signalling pathways by binding to ethylene receptors (e.g. ETR1), we examined whether ethylene regulates primary root growth tolerance to Fe in such a way. Root growth in the ethylene-insensitive (ethylene-receptor) mutant *etr1-3* was only slightly sensitive to excess Fe compared with WT seedlings; consistent with this, the constitutive ethylene signalling mutant *ctr1-1* (a negative regulator of the ETR1-regulated ethylene signalling pathway) displayed increased root-growth tolerance (Fig. 5D). In further experiments with the double mutant line *eto1-1/etr1-3*, we found that *etr1-3* suppressed the root-elongation tolerance (Fig. 5D) characteristic of *eto1-1*.

### Inhibiting ethylene production leads to reduction of both cell elongation and cell division in roots exposed to excess Fe

**Fig. 6.**
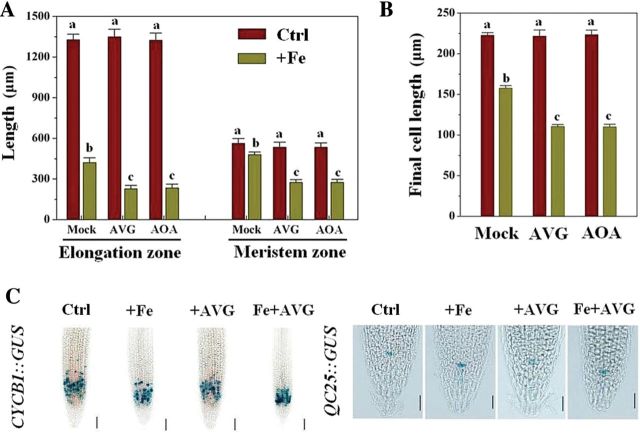
Effect of ethylene inhibitors on cell elongation and cell division of primary root tips under excess Fe treatment. Five-day-old WT seedlings were transferred to medium, and roots were supplemented with 350 μM Fe alone or in combination with 1 μM AVG or 50 μM AOA for 5 d, after which the data were analysed. (A) Root elongation and meristem zone sizes of 5-d-old *Arabidopsis* seedlings treated with 350 μM Fe alone or in combination with 1 μM AVG or 50 μM AOA for 5 d. Bars represent means±SE of nine or more plants. Different letters represent statistically different means at *P*<0.05 (one-way ANOVA analysis with Duncan post-hoc test). (B) Comparison of the effect of ethylene inhibitors on the final cell length in primary roots in the root-supplied Fe treatment (5 d). Values represent the means±SE of more than 80 cells from no fewer than seven independent plants. Different letters represent statistically different means at *P*<0.05 (one-way ANOVA analysis with Duncan post-hoc test). (C) Effect of ethylene inhibitors on the expression of *CycB1::GUS* and *QC25::GUS* after staining for 16h. One representative sample for each experiment is shown. Bars, 50 μm.

Given that excess Fe markedly inhibited root-tip cell elongation and cell division (Fig. 2), we asked whether endogenous ethylene levels in Fe-treated plants were related to cell elongation and cell division in root tips exposed to excess Fe. Exogenous AVG and AOA application had almost no effect on the length of the elongation and meristematic zones in the control (50 μM Fe), whereas they strongly reduced the length of both zones in the presence of excess Fe (350 μM Fe) (Fig. 6A). We then examined the length of differentiated cells just above the elongation zone. AVG and AOA application significantly reduced final cell length under excess Fe (50.5 and 52% inhibition, respectively) but had no effect in control roots (Fig. 6B). Furthermore, *CycB1::GUS* analysis showed that excess Fe in combination with AVG had no significant effect on the intensity of expression of *CycB1::GUS* in the root meristem compared with the control (*P<*0.05) (Fig. 6C and Supplementary Fig. S5C), but the extent and area of the GUS-stained region was reduced more than in the Fe/–AVG treatment (~57% reduction in combination vs ~33% alone of stained zone length, respectively) (Fig. 6C and Supplementary Fig. S5A, B). However, AVG application had little effect on the expression of *QC25::GUS* under excess Fe treatment (Fig. 6C and Supplementary Fig. S5D).

### Ethylene plays a role in modulating tissue Fe homeostasis under Fe stress, even without iron-plaque induction

To understand the function of iron-plaque formation under Fe stress in the context of primary root growth, we studied root-surface iron plaques in WT and mutant (*eto1-1*) using an optical microscope and SEM/EDX. SEM/EDX analysis showed that the weight percentage of Fe at the root surface was slightly altered in response to excess Fe treatment in both WT and mutant (Supplementary Fig. S6A at *JXB* online). However, under the Fe treatments used, the root surface of *Arabidopsis* WT and mutant showed no obvious (orange) plaques (Supplementary Fig. S6B).

**Fig. 7.**
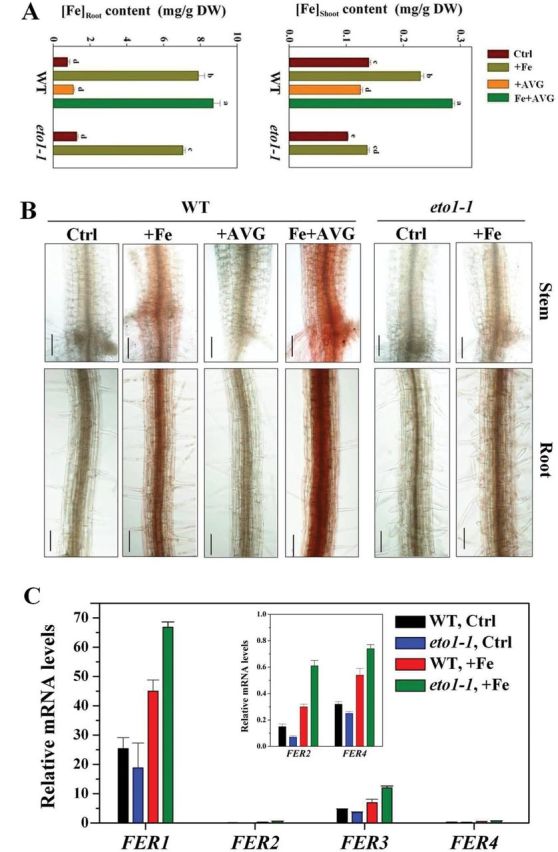
Effect of ethylene on tissue Fe homeostasis in excess Fe-treated seedlings. (A) Effect of ethylene on Fe contents in shoots and roots of *Arabidopsis* WT and *eto1-1* seedlings in root medium supplemented with 350 μM Fe alone or in combination with 1 μM AVG for 5 d. Values are the means±SE of three replicates. Different letters represent statistically different means at *P*<0.05 (one-way ANOVA analysis with Duncan post-hoc test). DW, dry weight. (B) Effect of ethylene on free Fe^2+^ concentrations in roots and stems of *Arabidopsis* WT and *eto1-1* seedlings in root medium supplemented with 350 μM Fe alone or in combination with 1 μM AVG for 4 d. Localization of ferrous Fe was analysed by 2,2′-bipyridine staining as described by Engel *et al.* (2012). Bars, 50 μm. (C) Expression of *FER1*, *FER2*, *FER3*, and *FER4* in roots of WT and *eto1-1* seedlings. Expression of *FER1*, *FER2*, *FER3*, and *FER4* were determined by quantitative real-time PCR after exposure of 5-d-old WT and *eto1-1* seedlings to 350 μM Fe for 6h. The relative mRNA level was normalized to *CBP20* expression. Values are means±SE of three replicates.

To examine whether and how ethylene regulates Fe accumulation and homeostasis in excess Fe-treated seedlings in absence of iron plaques, we first investigated the Fe content in root and shoot of seedlings grown *in vitro*, under control (50 μM Fe) and excess Fe (350 μM Fe) conditions, using ICP-AES. Fe content in both WT and *eto1-1* seedlings increased rapidly when exposed to excess Fe conditions, but Fe accumulation in WT seedlings, especially in shoots, was significantly greater than in *eto1-1* seedlings when cultivated under excess Fe (Fig. 7A). Under excess Fe, WT shoots and roots contained ~68 and ~14% more Fe than *eto1-1* mutants, respectively. Moreover, Fe content in excess Fe- and Fe+AVG-treated plants revealed that inhibiting ethylene production by AVG further increased the Fe content in WT, increasing by ~22 and ~11%, in shoot and root, respectively (Fig. 7A).

Free Fe^2+^ is the principal toxicity component under excess Fe toxicity (Becker and Asch, 2005). To determine whether ethylene modulates *Arabidopsis* tolerance by regulating root and stem free Fe^2+^ concentrations, we examined free Fe^2+^ concentrations in root and stem by staining in a mutant in the presence of an ethylene inhibitor. Fe^2+^ concentrations in both WT and *eto1-1* increased in roots and stem under excess Fe, especially in the vascular tissue and xylem (Fig. 7B), but much less so in the *eto1-1* mutant (Fig. 7B). Moreover, supplementation with AVG markedly increased free Fe^2+^ concentrations in Fe-treated WT compared with Fe treatment alone (Fig. 7B). In addition, we also utilized Perls’ stain to localize free Fe^3+^ in Fe-alone and Fe/+AVG-treated WT roots. Increased staining was observed in the roots of Fe-treated plants, and supplementation with AVG markedly increased the staining (Supplementary Fig. S7 at *JXB* online). Interestingly, we found that *eto1-1* seedlings showed slightly more pronounced Fe^3+^ staining than WT grown under excess Fe alone (Supplementary Fig. S7).

The expression of genes encoding ferritin (*FER1*, *FER2*, *FER3*, and *FER4*; Petit *et al.*, 2001) was also analysed by qRT-PCR. As shown in Fig. 7C, expression of ferritin genes was stimulated in both WT and *eto1-1*, and the induction of ferritin genes was significantly higher in *eto1-1* than in the WT under excess Fe. Similar to a previous report (Petit *et al.*, 2001), *Arabidopsis FER1* and *FER3* (especially *FER1*) were the two major ferritin genes expressed in roots in response to excess Fe (Fig. 7C).

### Ethylene is related to root K accumulation in Fe-treated seedlings

**Fig. 8.**
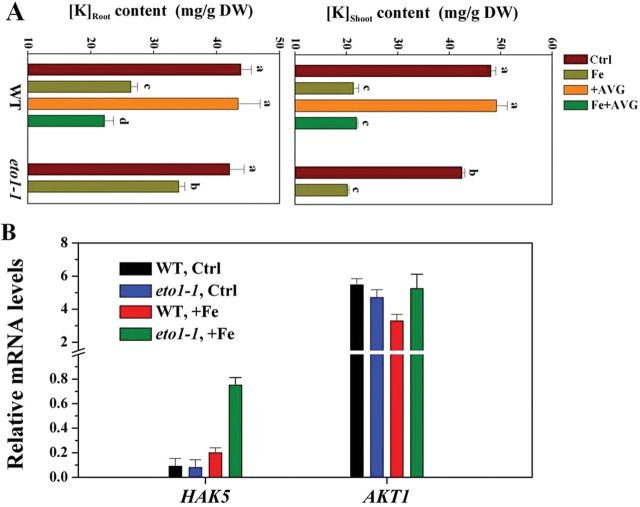
Effect of ethylene on tissue potassium (K) homeostasis in excess Fe-treated seedlings. (A) The effect of ethylene on K contents in shoots and roots of *Arabidopsis* WT and *eto1-1* seedlings in root medium supplemented with 350 μM Fe alone or in combination with 1 μM AVG for 5 d. Values are means±SE of three replicates. Different letters represent statistically different means at *P*<0.05 (one-way ANOVA analysis with Duncan post-hoc test). DW, dry weight. (B) Expression of *HAK5* and *AKT1* in roots of WT and *eto1-1* seedlings. Expression of *HAK5* and *AKT1* was determined by quantitative real-time PCR after exposure of 5-d-old WT and *eto1-1* seedlings to 350 μM Fe for 6h. The relative mRNA level was normalized to *CBP20* expression. Values are means±SE of three replicates.

Mineral content was also analysed in seedlings grown on control and excess Fe medium. ICP-AES analysis showed little difference in tissue mineral concentrations, including zinc, calcium, magnesium and manganese, between WT and *eto1-1* in response to excess Fe (Supplementary Fig. S8 at *JXB* online). However, a significant increase in root potassium (K) content was observed in *eto1-1* plants compared with WT (~30%). These differences were not observed in shoots (Fig. 8A). Furthermore, K content in shoots and roots of Fe- and Fe/+AVG-treated WT seedlings was determined. Supplementation with AVG reduced root, but not shoot, K content in Fe-treated seedlings, by ~20% (Fig. 8A). We further investigated the function of K in root growth under Fe stress. Primary root-growth suppression under excess Fe was alleviated by K^+^ addition (Supplementary Fig. S9 at *JXB* online), and *eto1-1* primary root growth appeared to be more tolerant to excess Fe under low K than WT (Supplementary Fig. S9).

The expression of two principal K^+^ transporter encoding genes, *HAK5* and *AKT1*, coding for root plasma membrane-resident high- and low-affinity K^+^ transporters, respectively (Szczerba *et al.*, 2009), in response to excess Fe was examined. *HAK5* expression was induced by excess Fe in both WT and *eto1-1* roots, but was ~6-fold more pronounced in *eto1-1* (Fig. 8B). In contrast to *HAK5*, transcript levels of *AKT1* were generally unresponsive to excess Fe treatment (Fig. 8B). The *eto2-1* mutant also displayed higher *HAK5* expression levels than WT in response to excess Fe (Supplementary Fig. S10 at *JXB* online).

## Discussion

Fe is both an essential micronutrient and is highly toxic when in excess, and inhibition of primary root growth is one of the chief symptoms of this toxicity (Becker and Asch, 2005; Fageria *et al.*, 2008). However, the mechanism underpinning this remains largely unknown. In the present study, we showed that excess Fe supplied to roots in *Arabidopsis* impacts both cell elongation and division, and this inhibition requires the root tip to be in direct contact with external excess Fe. We found that ethylene, but not ABA, is required for primary root tolerance to Fe toxicity. We further showed that ethylene can effectively regulate tissue Fe accumulation, even in the absence of iron-plaque formation. Our data revealed that ethylene supports tolerance to Fe by reducing free Fe^2+^ in the roots, stele, and xylem, and total Fe content in shoots. Additionally, ethylene also alleviates Fe-induced root K^+^ depletion. Thus, ethylene alleviates Fe’s inhibitory effect on *Arabidopsis* root growth by targeting tissue Fe and K^+^ homeostasis, not necessitating the induction of iron plaques.

We showed that the impact of Fe on root growth in *Arabidopsis* is not a systemic response. Split-plate results reported here provided a strong demonstration that physical contact of the primary root tip with Fe is necessary, and sufficient, for primary root inhibition (Fig. 2A, B), supporting the proposal that the root tip is the principal action site for Fe toxicity and resistance (Zhang *et al.*, 2011, 2012). Upon root-tip contact with excess Fe, we observed that cell elongation was suppressed dramatically, with cell division being reduced to a much lesser extent (Fig. 2C–E). From these observations, we conclude that root-supplied excess Fe arrests root growth primarily via decreasing cell elongation and division, and mainly results from the direct effects of external excess Fe upon the root tip. This is similar to what was observed under NH_4_^+^ toxicity, where root-tip contact was both essential and sufficient to effect NH_4_^+^ toxicity (Li *et al.*, 2010). In agreement with previous results, Fe deficiency significantly decreased *Arabidopsis* lateral root development, and increasing Fe supply improved lateral root number (Giehl *et al.*, 2012; Gruber *et al.*, 2013; Supplementary Fig. S2A), although a negative effect at 500 μM Fe was seen in the present study. The decrease in the lateral root number at 500 μM Fe possibly reflects the shorter length of the primary root, considering lateral root number is usually linked to primary root growth (Dubrovsky and Forde, 2012).

The present data indicated an enhanced ethylene evolution upon exposure of seedlings to excess Fe (Fig. 4A, B). Similar results have been reported in rice (Peng and Yamauchi, 1993; Majerus *et al.*, 2007*b*). However, unlike these previous studies, we here identified that expression of the genes encoding ACS and ACO, the two key enzymes responsible for ethylene synthesis, were transcriptionally upregulated by excess Fe (Fig. 4B, C). The enhanced expression of these genes was correlated with the observed Fe-induced ethylene production (Fig. 4A). To establish the links between Fe-induced ethylene production in roots and Fe-dependent root growth, both pharmacological and genetic approaches were employed. Remarkably, the excess Fe induced inhibition of primary root growth was alleviated in the presence of ACC, a precursor of ethylene, but inhibitors of ethylene biosynthesis (AVG and AOA; Peng and Yamauchi, 1993) aggravated the Fe-induced inhibition of root growth (Fig. 5A, B). The finding that mutants exhibiting ethylene-overproduction (*eto1-1* and *eto2-1*) showed increased root growth compared with WT under Fe stress (Fig. 5C) supports this notion. The observation that the externally root-supplied ethylene inhibitor AVG prevented the tolerance by elevated ethylene levels in *eto1-1* exposed to high Fe (Supplementary Fig. S4) further demonstrates the important role of ethylene for root growth under Fe stress. More specially, we found that ethylene inhibitors proved to significantly aggravate the Fe-induced inhibition of cell elongation and cell division (Fig. 6), consistent with the above-described root growth-suppressive phenotype induced by Fe (Fig. 2C–E). Based on these findings, excess Fe can inhibit root growth by reducing both cell elongation and division, and this inhibitory role is alleviated partially by ethylene. Our observations that *etr1-3* suppresses the root-growth tolerance characteristic of *eto1-1*, and inhibition of root growth in the constitutive ethylene signalling mutant *ctr1-1* in response to excess Fe was more than in WT (Fig. 5D), highlight the important roles the ETR1-CTR1-dependent ethylene signalling pathway play in Fe-dependent primary root growth.

There is ample evidence that formation of iron plaques on rice roots provides a physical barrier for influx of reduced iron, i.e. serves as an exclusion mechanism; meanwhile, ethylene is known to stimulate plaque formation (Becker and Asch, 2005; Majerus *et al.*, 2007*a*; Harahap *et al.*, 2014). Thus, the possible role of iron plaque in ethylene-regulated *Arabidopsis* root growth in response to Fe toxicity was investigated in our study. However, we did not find a significant influence of excess Fe on visible iron-plaque formation in *Arabidopsis* WT or *eto1-1* in this study (Supplementary Fig. S6). The form of Fe supply may be a potential explanation for the absence of visible iron plaques. In the present study, we supplied Fe in the form of Fe-EDTA based on the previous report that Fe-EDTA could maintain sufficient Fe stress over several days (Elec *et al.*, 2013), and Fe-EDTA as an Fe source has been reported not to form visible iron plaques as readily (Taylor *et al.*, 1984). Alternatively, no visible formation of iron plaques may in part also be the result of root washing inherent in the experimental protocols. However, roots were washed gently with deionized water in this study, and it has been reported that only the application of dithionite-citrate-bicarbonate (DCB) thoroughly removes iron plaques (McLaughlin *et al.*, 1985). One previous report showed that, for root surfaces without iron plaques, Fe was difficult to detect by SEM-EDX at the surface, although the total Fe content (DCB-extracted) was high in roots (Mi *et al.*, 2013), similar to the present SEM-EDX result (Supplementary Fig. S6A). Furthermore, deionized water washing could remove Fe adhering to the root surface (McLaughlin *et al.*, 1985), while SEM-EDX most effectively images root epidermal cells.

We furthermore observed over this time period, i.e. in the absence of plaque formation, that ethylene decreased Fe accumulation in roots (Fig. 7A). It is not known as this time whether ethylene can directly modulate root Fe influx and/or efflux, and more research is warranted to examine this. However, resistance to Fe toxicity is associated not just with tissue total Fe content but also with tissue free Fe^2+^ content. The accumulation of free Fe^2+^ in tissues is widely considered to be critical to the tolerance of Fe toxicity (Becker and Asch, 2005; Engel *et al.*, 2012). Under Fe toxicity, excess Fe-induced ethylene clearly decreased root- and stem-xylem Fe^2+^ levels (Fig. 7B). Furthermore, homeostatic regulation of shoot iron levels is necessary, and excess iron in shoots is considered to be the leading cause of whole-plant toxicity (Audebert and Sahrawat, 2000; Fageria *et al.*, 2008). In the present study employing both ethylene mutants and inhibitors, we showed that ethylene significantly decreased Fe accumulation in shoots (Fig. 7A). Although the mechanism by which endogenous ethylene regulates free Fe^2+^ and shoot Fe content under excess Fe remains to be defined, our hypothesis was that ethylene influences ferritin (*FER*) gene expression in roots (Fig. 7C). *FER1* and *FER3* are the two major ferritin genes expressed in *Arabidopsis* roots in response to excess Fe (Petit *et al.*, 2001; Fig. 7C), and iron homeostasis depends critically on these, with ferritin being able to sequester thousands of Fe^2+^ ions per molecule (Connolly and Guerinot, 2002), and these are, thus, critical in regulating tissue free Fe^2+^ concentration (Majerus *et al.*, 2007*a*, 2009) and in protecting roots against Fe-mediated oxidative stress (Reyt *et al.*, 2014) and may also affect shoot Fe accumulation as a result of enhanced Fe sequesteration in roots. Furthermore, Fe^2+^ decreases may occur in association with an alkalinization of apoplastic pH (Kosegarten *et al.*, 2004), which reduces Fe^2+^ mobility and chemical stability (Becker and Asch, 2005), and has been report to be caused by ethylene (Staal *et al.*, 2011). The (albeit slight) increase in Fe^3+^ staining in *eto1-1* mutant roots supports this possibility (Supplementary Fig. S7). Alternatively, *AtFRD3*, encoding a protein of the MATE (multidrug and toxin efflux) family responsible for the xylem loading of citrate, a potent Fe chelator, which is required for the correct distribution of Fe throughout the plant tissues (Durrett *et al.*, 2007), may be involved in ethylene-regulated Fe homeostasis under excess Fe condition. However, in the present study, we found that WT and *eto1-1* plants displayed similar levels of expression of *AtFRD3* under control conditions and that transcript levels were generally unresponsive to excess Fe treatment (Supplementary Fig. S11B). Furthermore, previous observations that *FRD3* was expressed weakly in Fe-sufficient roots (Inoue *et al.*, 2004; García *et al.*, 2010) also seem to discount this possibility. An additional mechanism examined here was that of the critical role of maintenance of K^+^ homesostasis to support root growth under Fe toxicity (Li *et al.*, 2001; Çelik *et al.*, 2010; Supplementary Fig. S8). Mutants with increased ethylene production showed less root K^+^ depletion under excess Fe and were more tolerant to excess Fe under low K^+^ (Fig. 8A and Supplementary Fig. S9). The Fe tolerance conferred by elevated ethylene levels and retention of higher root K^+^ may partially be linked to the elevated levels of transcripts encoding the high-affinity K^+^ transporter *HAK5* (*HIGH-AFFINITY K* ^*+*^ *TRANSPORTER5* (Szczerba *et al.*, 2009), known to be ethylene-responsive (Jiang *et al.*, 2013), observed in the *eto1-1* and *eto2-1* mutants (Fig. 8B and Supplementary Fig. S10). We speculate that elevated ethylene stimulates *HAK5* transcription to maintain root K homeostasis, considered important in controlling tolerance of primary root growth under Fe toxicity (Li *et al.*, 2001; Çelik *et al.*, 2010). Furthermore, a recent study by Reyt *et al.* (2014) observed that Fe homeostasis interfered with reactive oxygen species distribution in the primary root, and this interaction may contribute to primary root shortening under Fe excess. How precisely reactive oxygen species signalling and the ethylene pathway interact in root-growth acclimation to changes in Fe availability remains to be elucidated.

ABA, another important phytohormone, is often associated with stress, for instance, with Al toxicity (Hou *et al.*, 2010). However, ABA was shown not to be involved in root-growth responses under phosphorus deprivation (Trull *et al.*, 1997). In our study, application of ABA and the ABA-synthesis inhibitor FLU had no significant effect on primary root growth under Fe stress (Fig. 3). Furthermore, WT plants and ABA-deficient or -insensitive mutants showed essentially identical inhibition of primary root growth when exposed to varying concentrations of Fe. Thus, ABA is unlikely to be involved in the primary root inhibition under excess Fe toxicity. However, we currently cannot exclude that ABA may have an impact on other aspects of development in *Arabidopsis* under Fe toxicity. Al toxicity is another root growth constraint prevalent in acidic soils and often occurs in combination with Fe toxicity (Khabaz-Saberi *et al.*, 2010, 2012). However, in a previous study (Sun *et al.*, 2010), ethylene was reported to negatively impact root growth under Al tolerance. Mechanisms underpinning the differences in ethylene and ABA roles in response to Fe- and Al-mediated inhibition of root growth remain unknown, but might provide important clues to understanding the adaptive strategies of plants to excessive loads of both Fe and Al in acidic soils. Furthermore, moderately acidic pH values (pH >5; pH 5.3 in this study) have been reported to enhance Al rhizotoxicity by affecting plant cell membrane surface charge, as a result of which greater amounts of free Al can accumulate at the root-cell surface (Kobayashi *et al.*, 2013). According to calculations using Geochem-EZ (a multifunctional chemical speciation program; Shaff *et al.*, 2010), while most of the Fe is complexed with EDTA, small amounts of free Fe still exist (data not shown). Such insights gained from studying Al tolerance might offer useful clues to better understanding excess Fe toxicity.

In summary, our results showed that excess Fe contact with the root tip is both necessary and sufficient to the sensing of Fe stress in the root system of *Arabidopsis* and to the ensuing suppression of primary root growth, and results in a reduction of both cell elongation and cell division. Furthermore, we showed that elevated endogenous ethylene production results from upregulated expression of *ACS* and *ACO* genes, rather than ABA, and protects primary root growth under excess Fe toxicity, as demonstrated by aggravation of the inhibitory effect on root growth with ethylene antagonists and lower sensitivity of ethylene-overproduction mutants to excess Fe. Increased ethylene plays a positive role in tissue Fe homeostasis, even in the absence of iron-plaque formation. Ethylene reduces root Fe accumulation, increases the expression of genes encoding Fe-sequestering ferritin and minimizes stelar and xylem Fe^2+^ concentrations, thereby impacting both shoot Fe accumulation and toxicity within the root. In addition, ethylene positively impacts K^+^ homeostasis under Fe stress. Our results provide novel insight into how primary root growth is regulated in response to excess Fe stress. Further research into the mechanisms of interplay between excess Fe and ethylene evolution of plants will enable a fuller understanding of how plants respond to excess Fe stress by regulating hormonal signalling, and will be instrumental in the development of strategies to improve Fe toxicity tolerance in crops.

## Supplementary data

Supplementary data are available at *JXB* online.

Supplementary Fig. S1. Effect of pH on primary root growth in *Arabidopsis* (Col-0) when roots are supplied with or without excess Fe.

Supplementary Fig. S2. Effect on lateral root number and chlorophyll content in *Arabidopsis* when roots are supplied with Fe-EDTA.

Supplementary Fig. S3. Effect of excess Fe on the activity of the *EBS::GUS* in *Arabidopsis* root tissue.

Supplementary Fig. S4. Effect of AVG on *eto1-1* primary root growth under excess Fe stress.

Supplementary Fig. S5. Effect of excess Fe on the staining of *CycB1::GUS* and *QC25::GUS* in medium supplemented with or without AVG.

Supplementary Fig. S6. Analysis of iron plaques on the root surface of *Arabidopsis* WT and *eto1-1* mutants.

Supplementary Fig. S7. Fe^3+^ staining in roots of *Arabidopsis* WT and *eto1-1* seedlings.

Supplementary Fig. S8. Effect of ethylene on tissue mineral contents in excess Fe-treated seedlings.

Supplementary Fig. S9. Effect of exogenous K^+^ on primary root growth in WT and *eto1-1* seedlings.

Supplementary Fig. S10. The effect of excess Fe treatment on *AtHAK5* and *AtAKT1* transcript levels in *eto2-1* seedlings.

Supplementary Fig. S11. Effect of excess Fe on the expression of the *AtFRD3* gene and the specific analysis of primers used for RT-PCR.

Supplementary Table S1. Gene-specific primers used for RT-PCR.

Supplementary Data

## Abbreviations:

ABA: abscisic acid
ACC: 1-aminocyclopropane-1-carboxylic acid
ACO: ACC oxidase
ACS: ACC synthase
Al: aluminium
ANOVA: analysis of variance
AOA: amino-oxyacetic acid
AVG: aminoethoxyvinylglycine
EDX: energy-dispersive X-ray
Fe: iron
FLU: fluridone
ICP-AES: inductively coupled plasma atomic emission spectroscopy
K: potassium
QC: quiescent centre
qRT-PCR: quantitative real-time reverse transcription-PCR
SE: standard error
SEM: scanning electron microscopy
WT: wild type.
