# Supplementary material for: Ethylene is critical to the maintenance of primary root growth and Fe homeostasis under Fe stress in Arabidopsis
Source: J Exp Bot. 2015 Feb 22;66(7):2041–54. doi: 10.1093/jxb/erv005 (PMC4378635; doi:10.1093/jxb/erv005)
Supplement: Supplementary Data [file supp_66_7_2041__index.html]

Ethylene is critical to the maintenance of primary root growth and Fe homeostasis under Fe stress in Arabidopsis — Ethylene is critical to the maintenance of primary root growth and Fe homeostasis under Fe stress in Arabidopsis — Supplementary Data 

# Ethylene is critical to the maintenance of primary root growth and Fe homeostasis under Fe stress in *Arabidopsis*

## Supplementary Data

Data files

**Files in this Data Supplement:**

- Supplementary Data - Supplementary Data
