# Supplementary material for: Ethylene is critical to the maintenance of primary root growth and Fe homeostasis under Fe stress in Arabidopsis
Source: J Exp Bot. 2015 Feb 22;66(7):2041–54. doi: 10.1093/jxb/erv005 (PMC4378635; doi:10.1093/jxb/erv005)
Supplement: Supplementary Data [file supp_erv005_jexbot134841_file001.pdf]

Ethylene is critical to the maintenance of primary root growth and Fe homeostasis under Fe stress in *Arabidopsis*

Guangjie Li, Weifeng Xu, Herbert J. Kronzucker, Weiming Shi *

Supplementary Data

Supplementary data

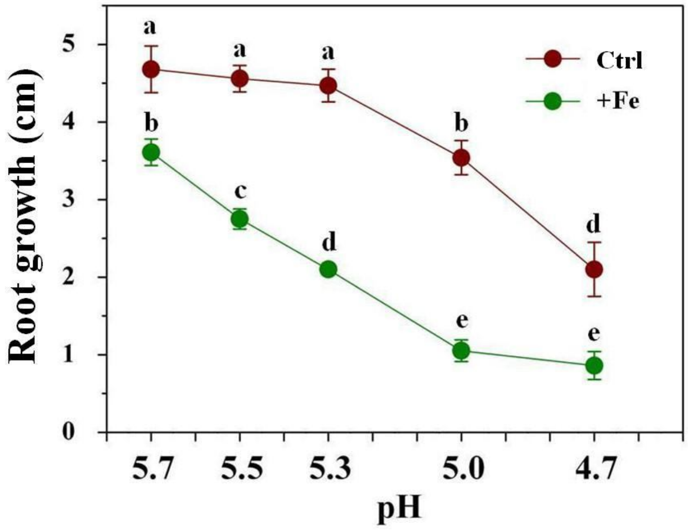

Supplementary Fig. S1 The effect of pH on primary root growth in *Arabidopsis* (Col-0) when roots are supplied with or without excess Fe. Seedlings at five days after germination (five DAG) were transferred to varying pH conditions in the root growth medium (bottom part) with or without Fe [provided as Fe-EDTA] and grown for an additional five days, after which root growth was measured. pH = 5.7 was used as the control. Data represent means of seven or more plants ± SE (error bars are not visible due to overlap with legend marks). Different letters represent means statistically different at the 0.05 level (one-way ANOVA analysis with Duncan post-hoc test).

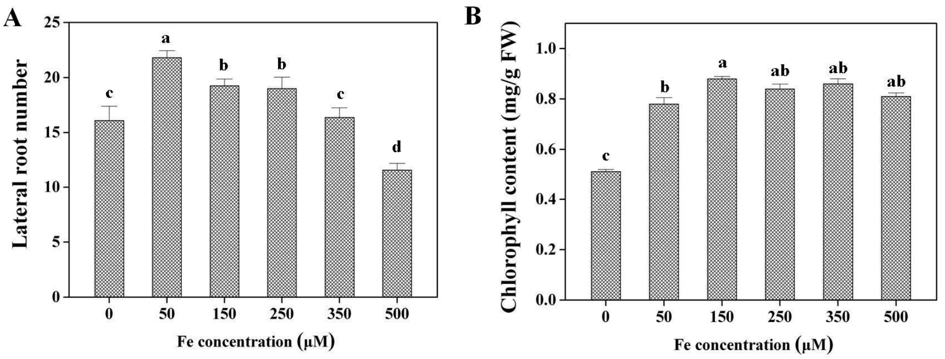

Supplementary Fig. S2 The effect on the lateral root number and chlorophyll content in *Arabidopsis* when roots are supplied with Fe-EDTA. Seedlings at five days after germination (five DAG) were transferred to serial concentrations of Fe-EDTA and grown for an additional five days.

(A) Lateral root number of five-day-old wild-type exposed to serial concentrations of Fe [provided as Fe-EDTA] for five days. Data represent means of eight or more plants ± SE.

(B) Chlorophyll content of five-day-old wild-type exposed to serial concentrations Fe for five days. Values are means ± SE of three replicates. Different letters represent means statistically different at the 0.05 level (one-way ANOVA analysis with Duncan post-hoc test). FW, fresh weight.

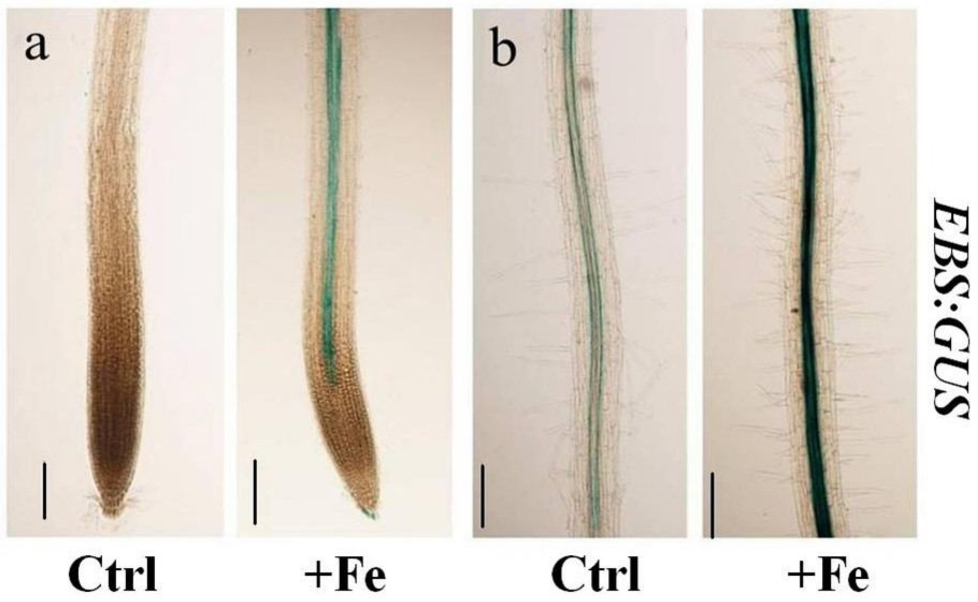

Supplementary Fig. S3 Effect of excess Fe on the activity of the *EBS::GUS* in *Arabidopsis* root tissue. Seedlings at four DAG were exposed to 350 μM in the roots for four days, and then ethylene reporter *EBS::GUS* activity was determined. One representative sample from each treatment (10 plants) is shown. Scale of bars = 100 μm. *a.* primary root tips. *b.* stele of primary root.

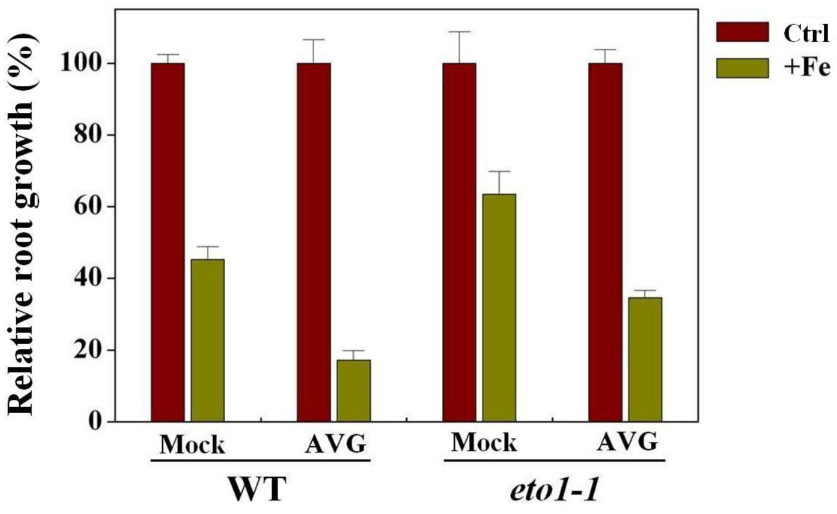

Supplementary Fig. S4 The effect of AVG on *eto1-1* primary root growth under excess Fe stress. Five-day-old wild-type and *eto1-1* seedlings were transferred to medium roots supplemented with 350 μM Fe alone or in combination with 1 μM AVG for five days. Values are the means ± SE, n ≥ 5. Primary root growth in wild type in control under mock or AVG conditions was 5.3 ± 0.12 and 5.34 ± 0.35 cm, respectively. In *eto1-1*, in control under mock or AVG conditions, it was 3.08 ± 0.27 and 3.84 ± 0.11 cm, respectively. Different letters represent means statistically different at the 0.05 level for a given genotype (one-way ANOVA analysis with Duncan post-hoc test).

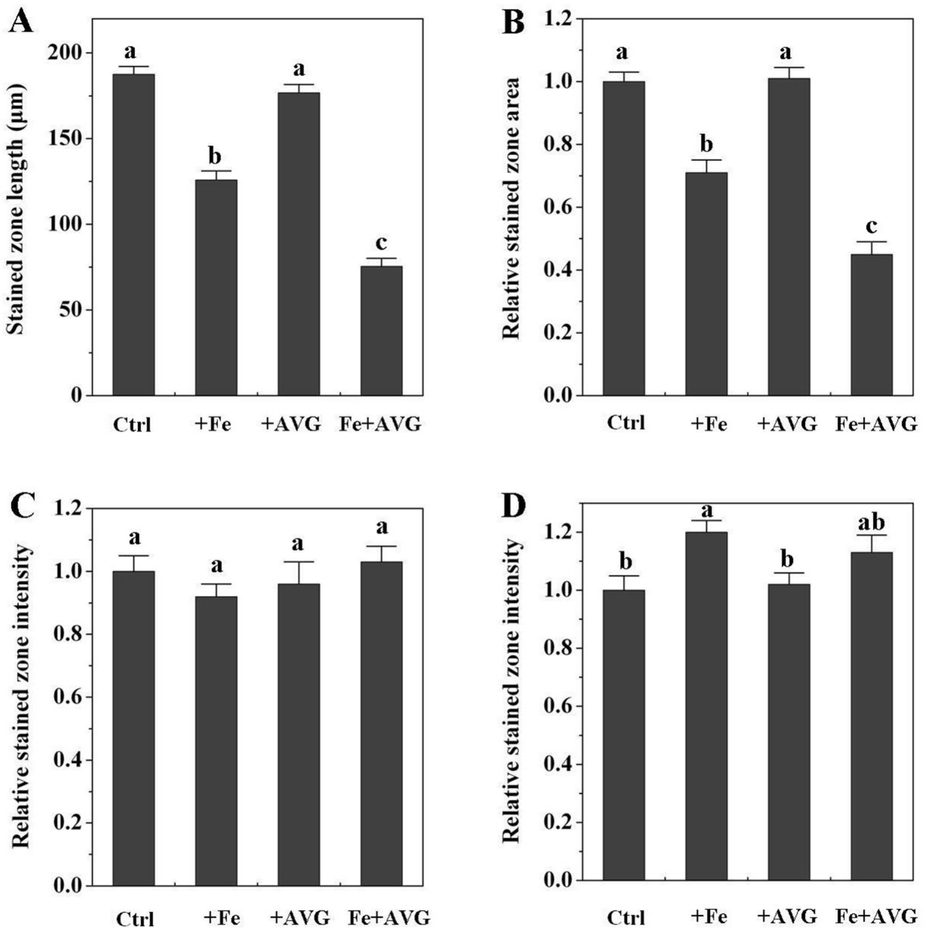

Supplementary Fig. S5 Effect of excess Fe on the staining of *CycB1::GUS* and *QC25::GUS* in medium supplemented with or without AVG. Five-day-old wild-type seedlings were transferred to medium roots supplemented with 350 μM Fe alone or in combination with 1 μM AVG for three days. (A) to (C) *CycB1::GUS* staining; (D) *QC25::GUS* staining. Values are the means ± SE, n = 5. Different letters represent means statistically different at the 0.05 level (one-way ANOVA analysis with Duncan post-hoc test).

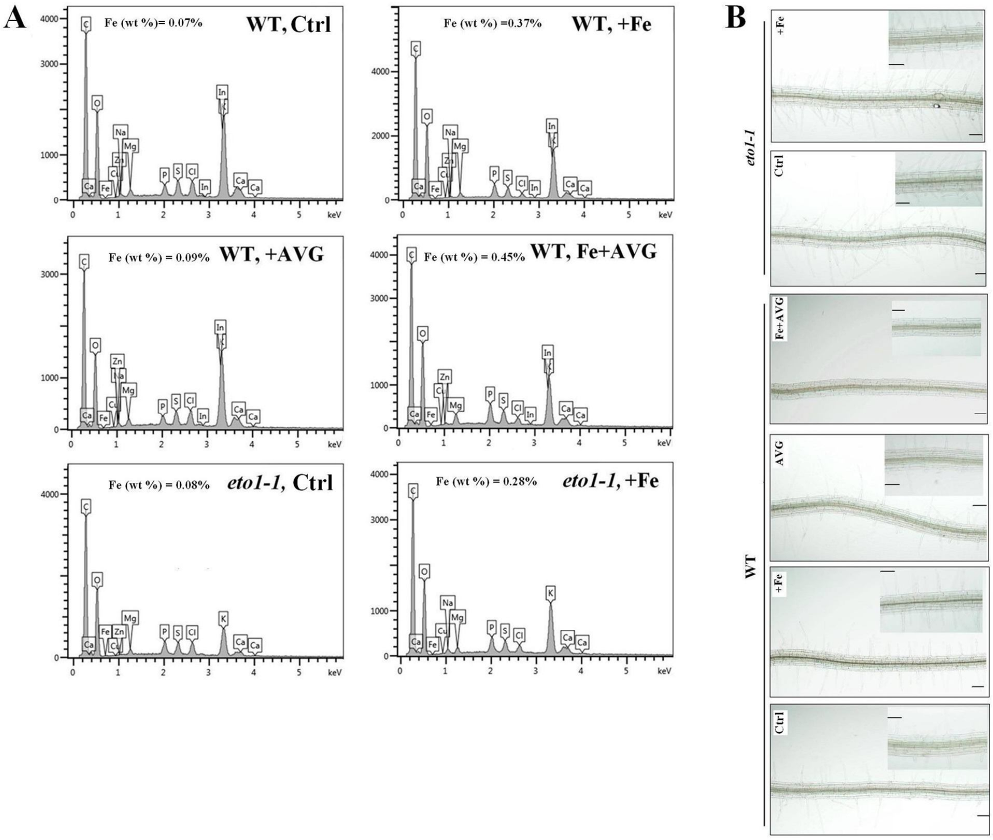

Supplementary Fig. S6 Analysis of iron plaque formation on the root surface of *Arabidopsis* WT and *eto1-1* mutant. Five-day-old wild-type and *eto1-1* seedlings were transferred to control or excess Fe medium with or without AVG for an additional five days of growth, and then the materials formed on the *Arabidopsis* roots were observed and analyzed using an optical microscope or SEM/EDX.

(A) Energy-dispersive X-ray spectra (EDX) of the *Arabidopsis* root surface as described by Mi *et al.* (2013). More than seven independent seedlings were analyzed. wt%, weight percentage (%) of the Fe in the root surface.

(B) Optical microscope images of *Arabidopsis* roots. One representative sample for each experiment is shown. Scale bars = 100 μm.

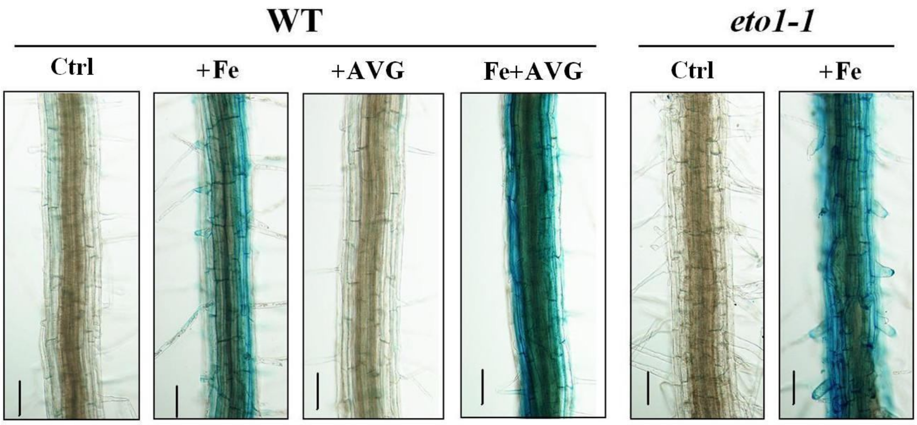

Supplementary Fig. S7 Fe^{3+} staining in roots of *Arabidopsis* WT and *eto1-1* seedlings. Five-day-old wild-type and *eto1-1* seedlings were transferred to medium roots supplemented with 350 μM Fe alone or in combination with 1 μM AVG for four days. Localization of ferric Fe was analyzed by Perl's staining as described by Stacey *et al.* (2008). Scale bars = 50 μm.

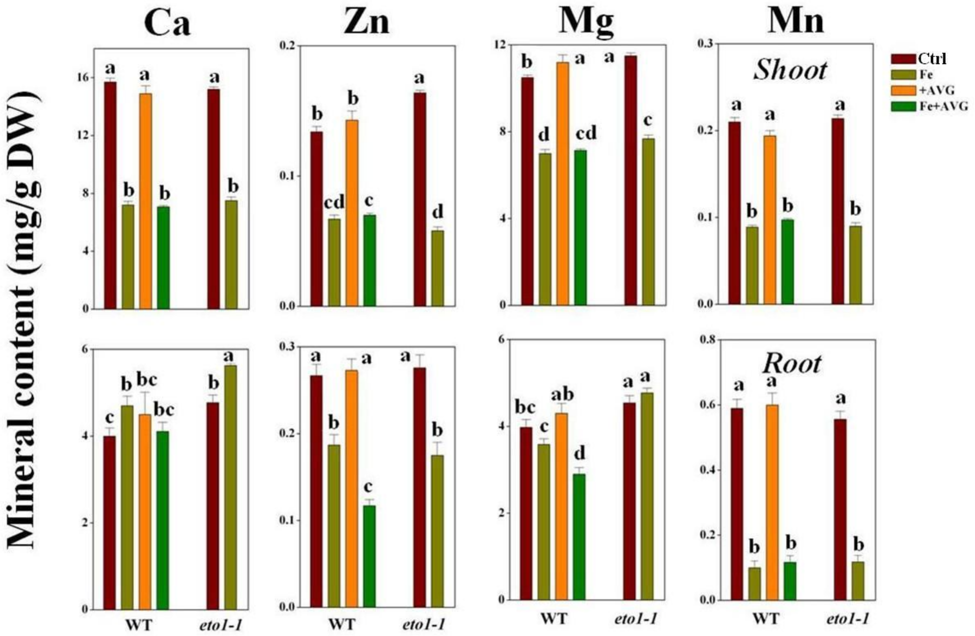

Supplementary Fig. S8 Effect of ethylene on tissue mineral contents in excess Fe-treated seedlings. The effect of ethylene on mineral contents in shoots and roots of *Arabidopsis* WT and *eto1-1* seedlings in roots supplemented with 350 μM Fe alone or in combination with 1 μM AVG for five days. Values are the means ± SE of three replicates. Different letters represent means statistically different at the 0.05 level (one-way ANOVA analysis with Duncan post-hoc test). DW, dry weight.

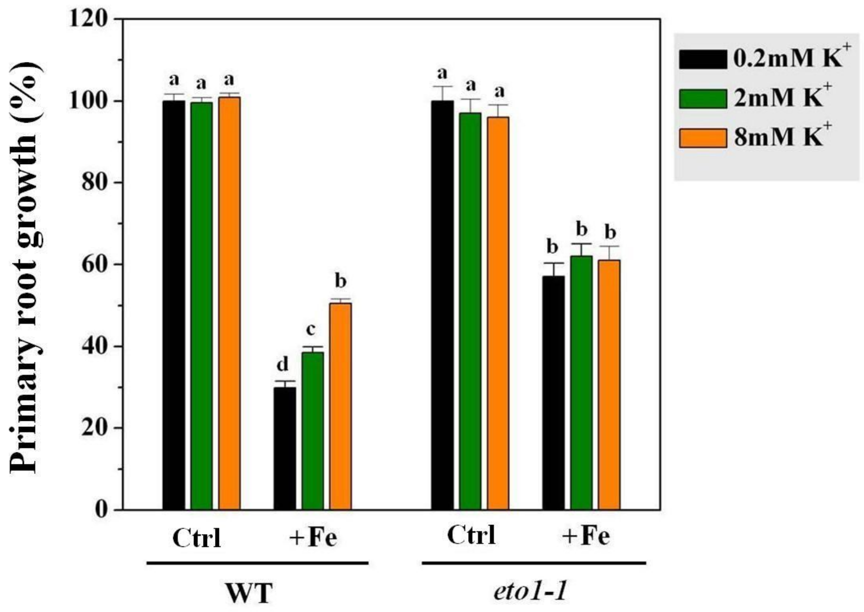

Supplementary Fig. S9 The effect of exogenous K^+ on primary root growth in wild-type and *eto1-1* seedlings. Five-day-old wild-type and *eto1-1* seedlings were transferred to medium supplemented with 350 μM Fe plus varying K^+ [provided as K_2SO_4] concentrations in the root medium. Values are the means ± SE, n ≥ 7. Primary root growth in wild-type and *eto1-1* in control was 5.3 ± 0.2 and 3.03 ± 0.24 cm, respectively. Different letters represent means statistically different at the 0.05 level for a given genotype (one-way ANOVA analysis with Duncan post-hoc test).

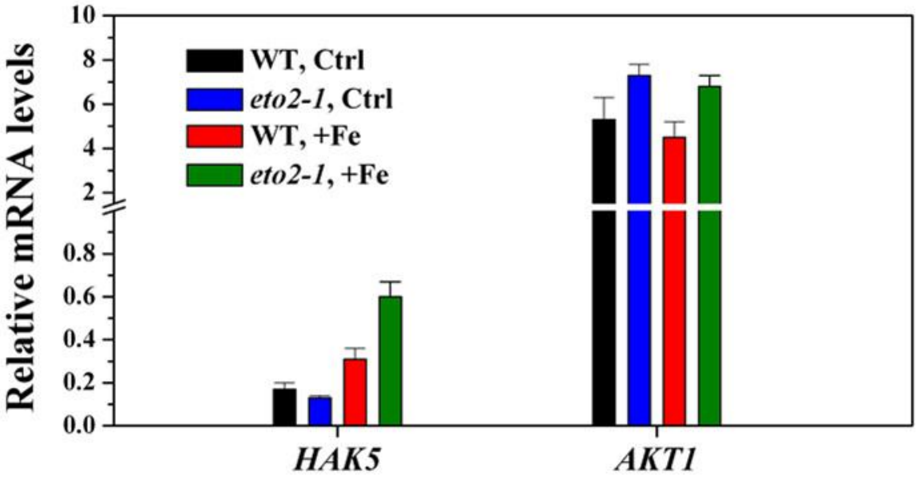

Supplementary Fig. S10 The effect of excess Fe treatment on *AtHAK5* and *AtAKT1* transcript levels in *eto2-1* mutants. Expression of *HAK5* and *AKT1* were determined by quantitative RT-PCR after exposure of five-day-old WT and *eto2-1* seedlings to 350 μM Fe for 6 hours. The relative mRNA level was normalized to the *CBP20* expression. Values are means ± SE of three replicates.

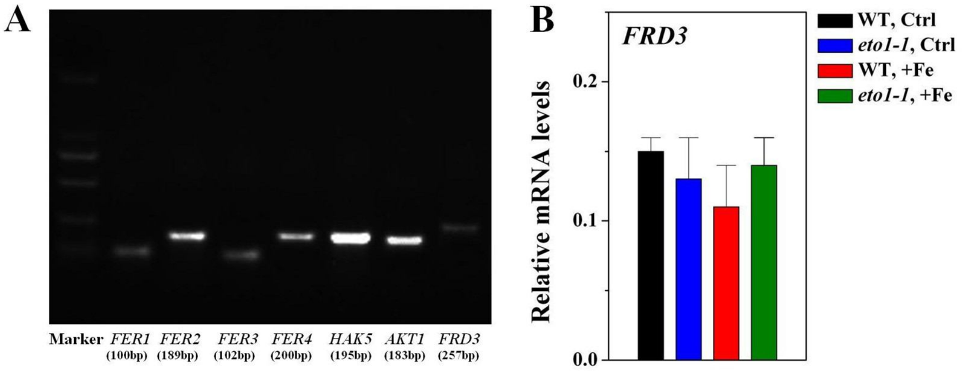

Supplementary Fig. S11. Effect of excess Fe on the expression of *AtFRD3* gene and the specific analysis of primers used for RT-PCR.

(A) The specific analysis of primers used for RT-PCR. Primer specificity was confirmed by ordinary PCR, as follows: 94°C for 5 min followed by 35 cycles of 94°C for 20 s, 60°C for 20 s, and 72°C for 30 s.

(B) Expression of *FRD3* was determined by quantitative RT-PCR after exposure of five-day-old WT and *eto1-1* seedlings to 350 μM Fe for 6 hours. The relative mRNA level was normalized to the *CBP20* expression. Values are means ± SE of three replicates.

Table S1 Gene-specific primers used for RT-PCR.

Gene	Forward Primers (5'-3')	Reverse Primers (5'-3')
HAK5	CGAGACGGACAAAGAAGAGGAACC	CACGACCCTTCCCGACCTAATCT
AKT1	GAGGAGTATCCAATGACCTGCTTT	TGTTCCAGTATCTACATCCACCAA
FRD3	TTTGTCGGGCGTTTAGG	TGCTGTGGCTGGTTGGT
FER1	TCCCATTGGATGTAGCACGAG	GATTAGCGGCGGTGAAAGAC
FER2	CAGCTAAGAAGAATAGGAAAGGGTC	TTAAGTACACACTCACAAGCATTCG
FER3	CTTGCTCGTCATTTGTACTCCC	TGTAACTTGACCCTCCCACCAC
FER4	TTAACGAGCAGATCAATGTGGAAT	CACCACGTTTGTTCTGATACTCCA
ACS2	TCATGGGAAAAGCTAGAGGTGGAAG	TCAACGGTTAATTTGAAATTGTCGG
ACS7	CCTGGGTTCCGTGAAAACGCATT	CGTCGTTAGGATCGGCGAGAATGA
ACS8	TGGGGTGATTTACTCCAACGATGATT	GACACTCGATGCCTGCAGCCTCTAG
ACS11	CTGGTTTCGGGTCTAAAGGAAGCGG	AATGACACGATGAGCCTGGAGAGATGTT
ACO1	CCGTGTAATGACAGTGAAGCATGGAAG	TCTCAAGTCTGGGGCCTTTGTCTCC
ACO2	GGATGTCGGTTGCATCGTTTTA	TACGGCTGCTGTAGGATTCAGTTC
CBP20	ACCATCGGAAACGACAAAGAG	CTTCACCATCGTCATCGGAGT
